# Between Extremes: Health Effects of Heat and Cold

**DOI:** 10.1289/ehp.123-A275

**Published:** 2015-11-01

**Authors:** Nate Seltenrich

**Affiliations:** Nate Seltenrich covers science and the environment from Petaluma, CA. His work has appeared in *High Country News*, *Sierra*, *Yale Environment 360*, *Earth Island Journal*, and other regional and national publications.

Climate change is expected to have profound effects on weather patterns and temperatures worldwide in the coming decades, with serious implications for public health.^1^ Among the many ways in which global warming bears on human health,^2^^,^^3^^,^^4^^,^^5^^,^^6^ few are more readily apparent than the trend of increasing heat waves, which are often regarded as the deadliest of all natural disasters.^7^^,^^8^ And despite current and future adaptation efforts,^9^^,^^10^ the overall health burden of heat waves could grow as average temperatures continue their upward tick and extreme heat events become more frequent, severe, and long-lasting.^11^

**Figure:**
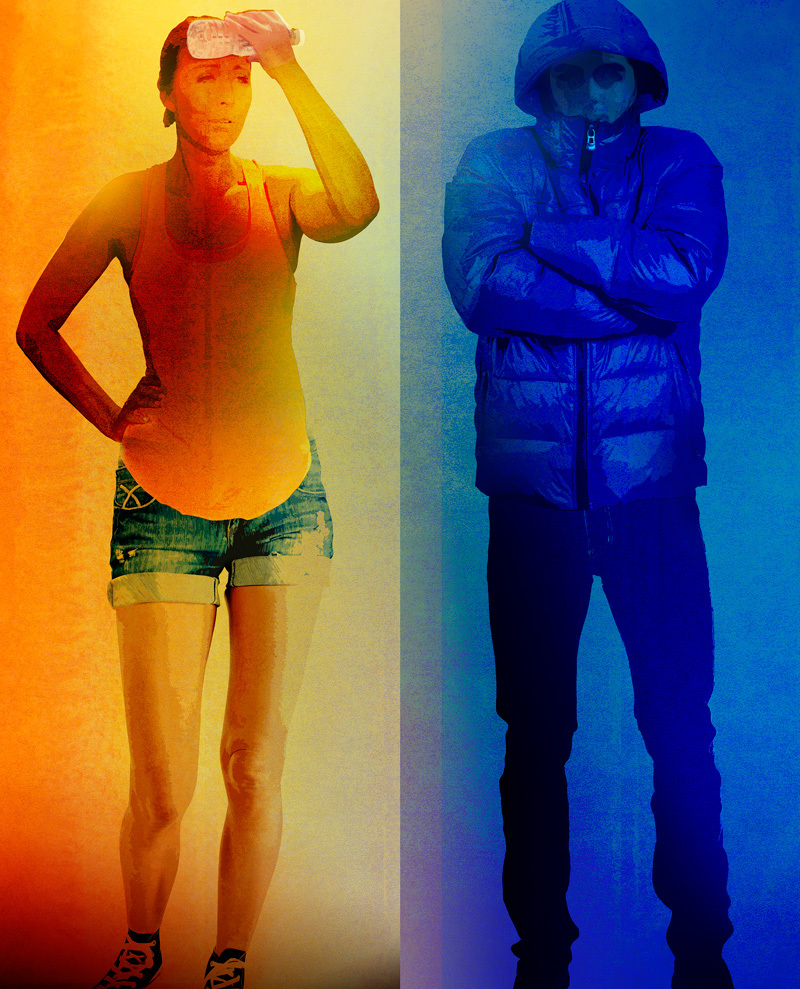
Although heat waves and cold snaps pose major health risks and grab headlines when they occur, recent studies have uncovered a more complex and perhaps unexpected relationship between temperature and public health. © Roy Scott

But while isolated heat waves pose a major health risk and grab headlines when they occur, recent research has uncovered a more complex and perhaps unexpected relationship between temperature and public health—on the whole, far more deaths occur in cold weather than in hot. This reality is obscured by the fact that, unlike heat-related health effects, which spike during discrete events, cold-related illnesses and deaths are diffuse throughout the year, don’t require extreme temperatures, and can lag well behind cold snaps.^12^^,^^13^

An analysis by the Centers for Disease Control and Prevention (CDC) of U.S. temperature-related deaths between 2006 and 2010 showed that 63% were attributable to cold exposure, while only 31% were attributable to heat exposure.^14^ In Australia and the United Kingdom, cold-related mortality between 1993 and 2006 exceeded heat-related mortality by an even greater margin—and is likely to do so through at least the end of the century.^15^ Researchers who evaluated 74 million U.K. and U.S. deaths reported in May 2015 that low temperatures are associated with 7.3% of all deaths versus just 0.4% for high temperatures, a ratio of more than 18 to 1.^16^

“You try to focus on the high-exposure situations, and sometimes you don’t realize that the important impact can be much higher for mild exposures, not because they are more dangerous but because they are far more common,” explains Antonio Gasparrini of the London School of Hygiene and Tropical Medicine, lead author of the 2015 paper.^16^ “These results are quite surprising because we never address [the relationship of temperature and health] from this perspective.”

In an effort to better understand the direct effects of cold temperature on human health in an era of climate change, researchers are fine-tuning their models and methods to account for nuances including seasonal public-health trends unrelated to the direct effects of temperature. One recent paper argued that cold-season deaths likely are largely associated with increases in respiratory infections such as influenza,^17^ thought to be facilitated both by cooler, drier air and by more hours spent indoors.^18^ In this view, future climate warming may bring less relief from the increases in mortality associated with cold weather than previously thought.

Other recent research has examined links between public health and extreme temperatures (highs and lows alike), which are more likely to actually cause effects, not just be associated with them. One study concluded that physiological acclimatization to extreme heat is unlikely to keep pace with climate change.^19^ Another helped quantify the so-called mortality displacement effect—deaths “bunch up” after a heat or cold event as seriously ill individuals die days or weeks earlier than they otherwise would have, then dip below normal.^20^

But evolving technological and behavioral adaptation to extreme temperature by individuals and communities is a more difficult concept to integrate into models, says Weiwei (Vivian) Yu, an expert on statistical modeling and methods at the University of Queensland. “We’re not sure how to measure these factors, even though we know they’re very important to the effect on health,” she says.

Ultimately, despite their huge scope of impacts, heat and cold exposures are individual phenomena and thus highly relative, researchers have found—from one person, home, neighborhood, city, and region to the next.^21^^,^^22^^,^^23^^,^^24^ That simple fact, it turns out, may dictate how researchers and public officials talk about temperature and health as climate change progresses.

## Cold, Heat, and the Human Body

Cold and heat can impair the human body and its physiological processes in innumerable ways, while also interacting with pre-existing conditions and chronic diseases. With both exposures, the primary concern is alteration of the body’s core temperature beyond a healthy range.

High body temperature is associated with increased heart and respiratory rates^25^ and, at extreme levels, damage to the brain, heart, lungs, kidneys, and liver.^26^ Blood vessels dilate near the skin, which can be problematic for those with heart issues, while the kidneys become stressed through a variety of pathways and may fail.^27^

“Heat waves exacerbate all sorts of chronic disease issues,” says Jeremy Hess, an emergency medicine physician and associate professor at the University of Washington. “The stresses you see on a population level depend on the underlying disease problems in that population.”

Heat stroke is a significant factor in the high mortality rates seen with heat waves. It contributes to a cascade of dangerous physical and mental effects and has historically been fatal in one in four people in which it is diagnosed, Hess says. However, he adds, fatalities seem lower in recent years, probably due to improved recognition and early medical treatment. Clinically speaking, the cutoff for heat stroke is a core temperature of 104°F (40°C); below that, the technical term is heat exhaustion, a similar, though far less severe condition.^28^

One uncertainty around the physiology of heat exposure involves the respiratory system, says Meredith McCormack, a pulmonologist at The Johns Hopkins University. “There is still a lot to learn about the physiologic responses that make individuals with underlying lung disease more susceptible to heat exposure,” she says. For instance, does the risk come from a systemic reaction to heat that exacerbates chronic respiratory diseases? Or is the effect of heat mediated by airway hyper-responsiveness that results from breathing hot air?^29^ More work is needed to tease apart the mechanisms.

In cold weather, the body can lose heat faster than it is produced, which uses up stored energy and can lead to hypothermia, defined as a core temperature below 95°F (35°C). Low temperatures cause veins and arteries to narrow and blood to become more viscous, increasing cardiac workload and leading to many of the same cardiovascular stresses as heat.^30^ “In true hypothermia, this extra cardiac workload is coupled with a host of other concerns, including increased cardiac muscle sensitivity that can lead to dysrhythmias,” Hess says. In addition to straining the heart and other organs, impaired blood flow and decreased metabolic activity due to low temperatures can affect the brain, making the victim unable to think clearly or move well.^31^

Hypothermia is most likely at extremely low temperatures, but it’s also possible well above freezing if a person becomes chilled from rain, sweat, or immersion in cool water.^32^^,^^33^ Hess says many hypothermia diagnoses occur in tandem with other illnesses and environmental exposures; some patients, for example, have systemic infections that disrupt thermoregulation and allow sepsis-related hypothermia to occur even in the summer.

The recent CDC study of U.S. temperature-related deaths found that for heat-related deaths, exposure to heat was the most frequently cited underlying cause of death on the death certificates, followed by heart disease and unintentional injuries. For cold-related deaths, the most frequently cited underlying cause of death was exposure to excessive cold, followed by unintentional injuries and heart disease.^14^

Generally speaking, people most at risk of illness or death from exposure to high or low temperatures include those less able to regulate their body temperature due to age, those with pre-existing conditions or chronic diseases, and users (especially heavy users) of alcohol or drugs.^14^^,^^34^ Individual vulnerability to heat and cold has also been found to vary with sex and race.^35^

Yet as much as we know about the physiological impacts of temperature exposures, without proper context there are few clear-cut ways of differentiating them in real time from those of other potential causes, and no clear fingerprints left behind for diagnosis after the fact. Both heat stroke and hypothermia can look like severe infection, Hess says, and afterward symptoms become even more contextual. That’s particularly true if the patient is deceased—“It’s difficult to ascertain a cause of death when you don’t have a core body temperature,” notes George Luber, associate director for climate change at the CDC.

Cardiorespiratory outcomes, in particular, are nonspecific and have many other causes and triggers besides heat and cold, says Diana Petitti, an independent consulting epidemiologist. “Therefore,” she explains, “whatever excess events there are due to temperature can get lost in the general background level.” Often, heat and cold are simply overlooked as factors in deaths. As a result, temperature-related health outcomes, which by many measures already surpass those due to all other weather phenomena, are almost certainly under-reported—potentially by a lot.

**Figure:**
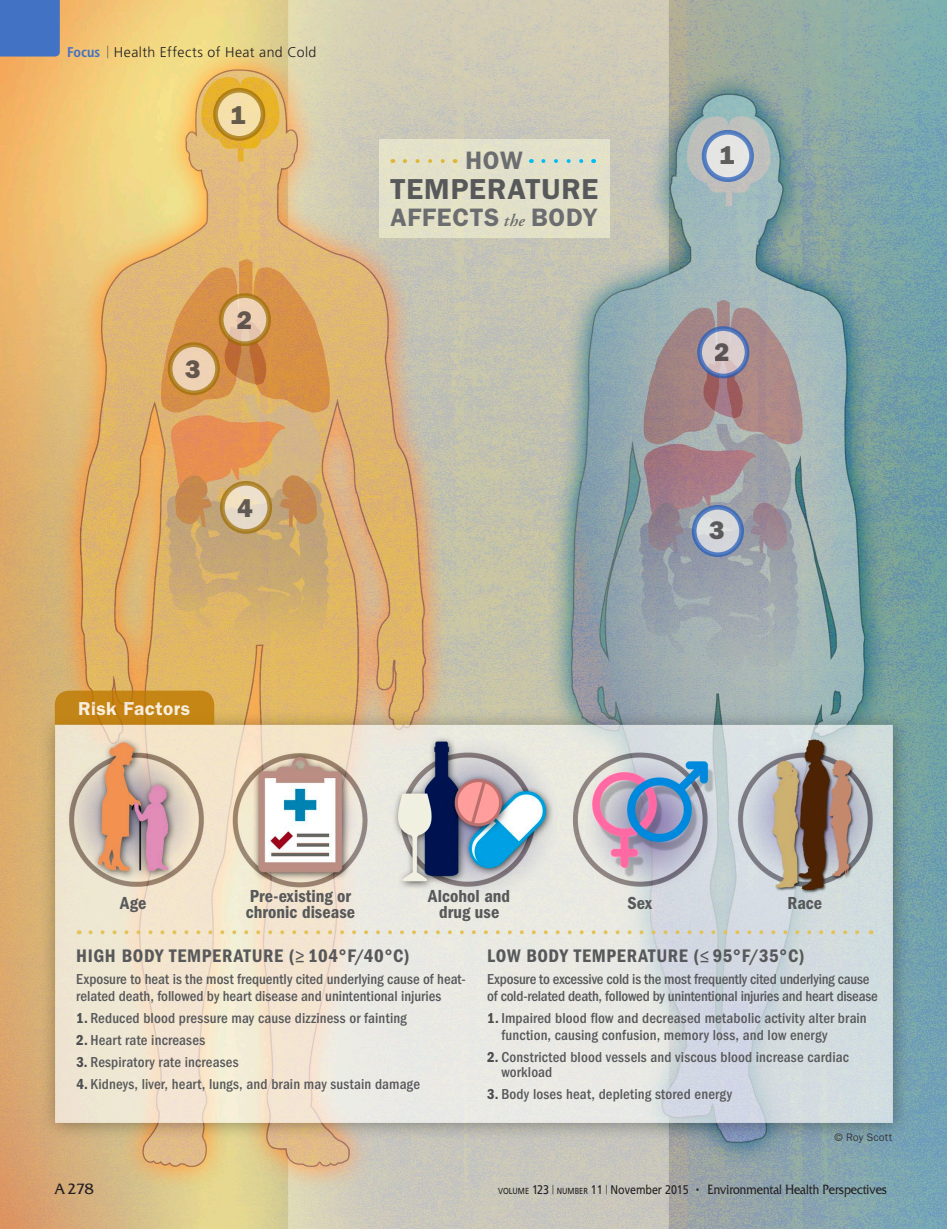


## Other Risk Factors

The outdoor environment is not the sole determinant of temperature-related health risks. When weather conditions are harsh, qualities of our cities, neighborhoods, homes, and individual behavior can have a huge influence on how we experience them, both for better and for worse.^15^^,^^36^^,^^37^^,^^38^

In a 2014 commentary, Colorado State University researcher Brooke Anderson showed how logistical lapses—including summertime vacations among city leaders and health workers, impaired infrastructure from power outages and melted roadways, insufficient supplies including fans and air-conditioning units, and overwhelmed public services and cooling centers—all conspired to exacerbate health impacts in three of modern history’s deadliest heat waves: Chicago 1995, France 2003, and Russia 2010.^39^

Lower-income areas may have fewer resources to prepare for and adapt to extreme weather events. When the CDC compared heat-related impacts across different categories of urbanization and income, it found that in general, weather-related death rates (including those from floods, storms, and lightning) were 2–7 times higher in low-income counties than in high-income counties.^14^

The study also showed that heat-related death rates were generally higher in the most urbanized areas than in less urbanized areas, although some of the highest rates occurred in rural areas of the South and West.^14^ For city dwellers, this may be a function of the urban heat-island effect, in which densely built-up areas absorb more heat during the day and release it more slowly at night.^40^ The high number of heat-related deaths in some rural areas was one of the study’s more surprising and interesting results, says lead author and CDC biostatistician Deborah Ingram, even if the nature of the relationship isn’t yet clear. As expected, the researchers also found that cold-related deaths were highest in the most rural areas nationwide, particularly the West.^14^

Among other variables influencing the public health impacts of high heat, one of the simplest is often regarded as the most powerful: air conditioning. Phoenix, Arizona, sees daytime highs near or above 90°F (32.2°C) for most of the year, and above 100°F (37.7°C) for the rest. Yet a recent study counted only 424 heat-related deaths in the city during the hottest part of the year (May 15 through October 15) over the period 2000–2011, an average of just 35 deaths per year. By contrast, with temperatures that rarely exceeded what is considered normal during a Phoenix summer, the 2003 European heat wave killed more than 70,000 people in three months.^41^

The difference is due in part to acclimatization, but it’s also worth considering that while air conditioning is both rare and unpopular across Europe despite rising temperatures,^42^ within the greater metropolitan Phoenix area 95% of occupied housing units have central air conditioning, the new paper reports.^41^ “[Phoenix is] a dangerous place to be if you don’t have access to cooling resources for almost the entire summer,” says coauthor David Hondula, a researcher at Arizona State University.

Hondula was lead author on a separate study designed to quantify the spatial variability of temperature-related outcomes within another heat-adapted city, Brisbane, Australia, where summers are routinely warm and humid. He and coauthor Adrian Barnett of the Queensland University of Technology found that, on average, areas with higher rates of heat-related hospital admissions had higher population densities and higher proportions of low-income residents.^43^ Five areas of the city, including three of the highest-income areas studied, actually had fewer hospitalizations associated with hot days. In at least two of these areas, the authors suggest this could be due to the protective effect of ample nearby green space and open water.

But there’s an interesting twist to Brisbane’s ability to cope with heat, says Barnett. The characteristic “Queenslander” architecture there involves elevating living spaces on stilts to maximize ventilation and capture summer breezes. However, during the mild winters, with average daily highs of 71–76°F (22–25°C) and lows of 50–56°F (10–14°C), this makes the homes harder to heat, potentially increasing cold-weather mortality. In fact, cold-weather mortality exceeds hot-weather mortality in Brisbane, as it does elsewhere in Australia.^17^

“There’s generally an underappreciation of how cold winters are, and some people even believing that it’s healthy,” says Barnett. “People in Scandinavia would never expose themselves to [Brisbane’s lowest] temperatures inside their homes.” Indeed, in much the same way that heat has proven more deadly in Europe, studies have confirmed that excess mortality due to cold weather is more pronounced in temperate regions than in cold regions, a fact blamed on, among other things, houses improperly designed for lower temperatures.^44^^,^^45^

## Interventions

As an intervention to reduce both cold- and heat-related deaths in Brisbane, Barnett recommends better insulation of its poorly sealed Queenslander homes. A 2007 study of 1,350 homes in New Zealand—which has a temperate climate cooler than that of Brisbane—found that better insulation in homes was associated with improved occupant health during the winter.^46^

But there’s less evidence to support another simple intervention, one that is typically the first line of defense against extreme heat in homes without air conditioning: electric fans. A 2012 Cochrane Review of studies addressing the ability of electric fans to reduce the health effects of heat waves found no studies that met its standards for research. Among the studies the reviewers evaluated but ultimately rejected as low quality, some claimed that fans alleviated health problems, while others suggested they worsened them^47^

Many efforts have been made to describe or review intervention efforts and adaptation in general,^48^^,^^49^^,^^50^^,^^51^^,^^52^ but as the electric fan example indicates, far fewer have reliably evaluated health outcomes tied to specific approaches.^53^^,^^54^ That being the case, Barnett is among those who believe the field should pivot from quantifying current health impacts toward testing solutions.

“Intervention studies are a lot more costly than epidemiological studies, but that’s where I feel the field should be going,” Barnett says. “It is a complex epidemiological situation, but potentially simple solutions might work.”

One example might be to distribute warm clothing during cold snaps—“a randomized control study of giving people [thermal underwear] would show what’s most effective,” Barnett suggests. Another might be to provide heating payments to low-income, high-risk populations, such as the British government is already doing,^55^ and then evaluate changes in health outcomes.

Marie O’Neill, an associate professor at the University of Michigan whose research group addresses sustainability and environmental justice, is passionate about connecting the epidemiology of cold and heat to interventions that offer more than one benefit. “A lot of the things we can do to be more resilient to the climate change that’s occurring are things that can have multiple impacts,” she says.

For example, building features such as cool roofs, passive heating and cooling, passive ventilation, and improved insulation may not only moderate temperature exposures but also improve indoor air quality in other ways, as well as reduce energy demands.^56^ One technique developed in the Middle East, O’Neill notes, involves pulling cool air from underground to circulate throughout a building—as in a geothermal heat pump—to improve occupant comfort with minimal energy use in one of the planet’s hottest climates.

Building design, city and neighborhood planning, and individual messaging designed to reduce the health effects of extreme temperatures will be key as climate change progresses, O’Neill says, yet the discussion must encompass other potential impacts, too. “It’s important to keep a holistic view on climate change and the variety of health consequences,” she says.

Understanding the many variables that influence temperature-related health outcomes and using them to design efficient interventions has become an imperative for the field, O’Neill believes. “Getting those research results applied is really critical,” she says. “To me, that’s a critical area for public health.”

Although heat and cold are currently associated with many more deaths than other weather phenomena, it’s uncertain if or how that may shift. That’s especially true since climate change is projected to trigger increasingly problematic flooding, drought, vectorborne disease transmission, agricultural disruption, energy shortages, air pollution, wildfires, sanitation challenges, human migration, violence, and conflict.^57^

Columbia University professor of environmental health sciences Patrick Kinney says it’s difficult to compare temperature against other health risks expected with climate change. “Heat impacts have been kind of like the low-hanging fruit for climate-health research to date,” he says. “We know that direct temperature effects are important, but we don’t yet know how to rank those effects among the full range of climate-induced health impacts.”

And while increased adoption of air conditioning throughout the world could well mitigate the effect of rising average temperatures on heat-related deaths,^58^ Kinney notes that this and other current adaptation trends may be counterbalanced by the aging—and thus increased vulnerability—of populations in Europe, the United States, Japan, and other parts of the world.
